# Editorial: Multiple Myeloma: Molecular Mechanism and Targeted Therapy

**DOI:** 10.3390/ijms25073799

**Published:** 2024-03-28

**Authors:** Despina Bazou, Paul Dowling

**Affiliations:** 1 School of Medicine, University College Dublin, D04 V1W8 Dublin, Ireland; 2 Department of Biology, Maynooth University, W23 F2K8 Kildare, Ireland

Multiple myeloma (MM) is a plasma cell disorder representing the second most common blood cancer [1]. MM is still defined as an incurable disease, but during the last 20 years, major progress has been made in the understanding of the pathophysiological mechanisms associated with MM. This expanded knowledge base has hastened the development of very successful therapeutics, including proteasome inhibitors, immunomodulatory drugs and monoclonal antibodies [2]. The development of such novel therapeutic options has yielded higher response rates and improved progression-free survival and overall survival rates.

Immune-based therapies, including Daratumumab, a humanized mAb that targets CD38, and Isatuximab, a chimeric mAb that binds to a different epitope of this cell surface receptor, have made an impact across all groups of MM patients [3]. Immunomodulatory drugs (IMiD) have been a principal element of the backbone of therapy for MM, starting with the first-generation IMiD Thalidomide, followed by the next-generation IMiD Lenalidomide and Pomalidomide [4]. Proteasome inhibitors (PIs) have been a highly influential therapeutic class in MM, a class that includes bortezomib, carfilzomib and ixazomib [5]. More recently, CAR-T cells, including Idecabtagene vicleucel and ciltacabtagene autoleucel, both of which target BCMA on the surface of myeloma cells, and bispecific antibodies, functioning by generating an immunologic link leading to the targeting of the malignant cell by the activated effector cell, are displaying beneficial anti-myeloma activity [6,7]. This exemplifies the range of new therapeutic approaches for the treatment of MM that have been developed and approved in recent years. However, alternative approaches are needed in triple-class refractory patients, which continues to be considered an important unmet clinical need.

The intention of this Special Issue is to highlight recent developments in our understanding of the molecular complexity of MM, strategies employed to identify new therapeutic targets and the identification of predictive biomarkers that will assist clinicians in determining which treatment approach is most likely to benefit individual patients (contribution 1).

New measures to stratify MM patients based on prognostic and predictive biomarkers are needed to manage patients based on their individual disease-related characteristics. ERBB2 (v-erb-b2 avian erythroblastic leukemia viral oncogene homolog 2, also known as HER2) is a gene that encodes for the receptor tyrosine-protein kinase erbB-2. ERBB2 has been found to be amplified in several cancers, including breast invasive ductal carcinoma, lung adenocarcinoma, colon adenocarcinoma and bladder urothelial carcinoma (contribution 2). Recently, it was discovered that patients with higher levels of ERBB2 mRNA in their malignant cells experienced significantly increased mortality, shorter progression-free survival and worse overall survival when evaluating 787 MM patients treated on contemporary standard regimens, using an RNA sequencing approach (contribution 3).

Investigating microRNA (miRNA) expression patterns in MM, hsa-miR-328-3p and hsa-miR-409-3p were found to be significantly downregulated in the early mortality group (contribution 4). A model consisting of hsa-miR-409-3p, hsa-miR-328-3p, age and R-ISS 3 achieved an area under the curve (AUC) of 0.863 using logistic regression analyses to assess the early mortality rate.

Cancer-associated thrombosis (CAT) is an important cause of morbidity and mortality for patients with malignancy. The dynamic interaction between platelets and myeloma cells opens up the possibilities of utilizing platelets for diagnostic and therapeutic purposes, as discussed by Kulkarni et al. (contribution 5).

Extramedullary multiple myeloma (or extramedullary disease, EMD) represents an aggressive form of multiple myeloma (MM), characterized by the ability of a clone and/or subclone to survive independent of the bone marrow microenvironment. McAvera and co-workers describe high-risk cytogenetic abnormalities and gene signatures associated with EMD, and the potential for some of these to contribute as disease biomarkers and potential therapeutic targets (contribution 6).

Clinical decision support (CDS) approaches have the ability to analyse significant amounts of data and advise on treatment approaches, increase efficiency and identify potential problems. Katsenou and co-workers compared several machine learning (ML) algorithms including Random Forest and support vector machines (SVM) to classify patients as sensitive/resistant to specific MM therapeutics (contribution 7). The results suggest that utilizing proteomics data (mass spectrometry derived) is a valid approach for identifying effective treatment options. The best-performing ML model was determined to be the SVM, providing an accuracy of 81, on average.

Currently, an accumulating body of evidence links autophagy to the process of drug resistance of various cancer types. Clavero and co-workers identified single-nucleotide polymorphisms (SNPs) in six loci, CD46, IKBKE, PARK2, ULK4, ATG5 and CDKN2A associated with MM risk, when analysis was performed on 234 autophagy-related genes (contribution 8). These SNPs were associated with alterations of specific immune cells and cytokine-dependent pathways.

Cancer cells develop multifaceted and dynamic microenvironments that affect their growth, invasion and metastatic potential. MM cells are located in bone marrow (BM) niches, providing a favourable environment for migration, proliferation and survival of MM cells, through their interaction with various tumour microenvironment-associated cells. Recently, inhibiting both protein kinase C (PKC) and nuclear factor kappa B (NF-κB) signalling pathways in BM mesenchymal stem cells (BM-MSCs) reduced cell survival in H929 (MM cell line) and increased its susceptibility to bortezomib (contribution 9). Increased levels of the serine protease uPA (urokinase-type plasminogen activator) and its receptor uPAR (CD87) have previously been associated with tumour progression and increased metastatic potential. Preliminary results showed that uPAR inhibition exerted a potent anti-inflammatory effect by almost abolishing IL-6 and DKK-1 secretion of primary MM-derived adherent cells, important molecules involved in disease progression and drug resistance (contribution 10).

Collectively, these studies highlight the different molecular mechanisms of MM development, identify viable novel targets for MM therapy and identify potentially valuable biomarkers for use as prognostic or predictive indicators.
